# Evidence that Transcription Factor AP-2γ Is Not Required for *Oct4* Repression in Mouse Blastocysts

**DOI:** 10.1371/journal.pone.0065771

**Published:** 2013-05-31

**Authors:** Inchul Choi, Timothy S. Carey, Catherine A. Wilson, Jason G. Knott

**Affiliations:** 1 Developmental Epigenetics Laboratory, Department of Animal Science, Michigan State University, East Lansing, Michigan, United States of America; 2 Department of Biochemistry and Molecular Biology, Michigan State University, East Lansing, Michigan, United States of America; The Babraham Institute, United Kingdom

## Abstract

In mouse blastocysts segregation of the inner cell mass (ICM) and the trophectoderm (TE) is regulated by the mutually antagonistic effects of the transcription factors Oct4 and Cdx2 expressed in the ICM and TE, respectively. In contrast, in other species such as bovine and human, Oct4 is not restricted to the ICM and continues to be expressed in the Cdx2-positive TE. A recent comparative study of the bovine and mouse *Oct4* promoters revealed that additional mechanisms might act in conjunction with Cdx2 to downregulate *Oct4* expression in the mouse TE lineage. For instance, the mouse *Oct4* distal enhancer contains an AP-2γ (Tcfap2c) binding motif that is absent in the bovine and human *Oct4* distal enhancer. Nonetheless, the functional relevance of Tcfap2c in *Oct4* repression during mouse preimplantation development was not tested. To elucidate the role of Tcfap2c in *Oct4* expression an RNA interference approach was utilized. Depletion of Tcfap2c triggered a decrease in *Oct4* expression at the 8-cell and morula stage. Remarkably, at the blastocyst stage depletion of Tcfap2c and/or its family member Tcfap2a had no effect on *Oct4* repression. To test whether Tcfap2c interacts with Oct4 to positively regulate *Oct4* expression, chromatin immunoprecipitation and *in situ* co-immunoprecipitation analyses were performed. These experiments revealed Tcfap2c and Oct4 binding were enriched at the *Oct4* distal enhancer in embryonic stem (ES) cells, but were rapidly lost during differentiation into trophoblast-like cells when *Oct4* became repressed. Moreover, Tcfap2c and Oct4 interactions were detected at the morula stage, but were lost during blastocyst formation. In summary, these data demonstrate that Tcfap2c is not required for *Oct4* silencing in mouse blastocysts, but may be necessary for the maintenance of *Oct4* expression during the 8 cell-to-morula transition. These findings support the notion Cdx2 is the predominant negative regulator of *Oct4* expression during blastocyst formation in mice.

## Introduction

During mouse preimplantation development dynamic expression patterns of the transcription factors (TFs) Oct4, Nanog, Sox2, Tead4, Gata3, and Cdx2 affect cell fate, and specify the first two lineages, the inner cell mass (ICM) and trophectoderm (TE) at the blastocyst stage [1]–[3]. Of these TFs, Oct4 (encoded by *Pou5f1*) is expressed uniformly in early cleavage stage embryos from oocyte to morula, and becomes confined to the ICM after blastocyst formation [4], [5]. In mouse embryos downregulation of Oct4 in the outer TE cells is strongly mediated by Cdx2 and the chromatin remodeling protein Brg1 (*Brahma-related gene 1*) [6]–[8]. However, in other species such as humans and cattle OCT4 is widely expressed in both the ICM and TE [9]–[11].

A recent study reported that the differences in *Oct4* expression among mice, humans, and cattle is attributed to the presence of an AP2 binding site (Transcription activating protein 2: Tcfap2 in mouse or Tfap2 in human and cattle) in the conserved region 4 (CR4) of the mouse *Oct4* distal enhancer region [12], [13]. For example, Berg and co-workers showed that a mouse *Oct4*-GFP transgene containing the CR4 regulatory region is restricted to the ICM in mouse blastocysts, but not in bovine blastocysts [12]. Reciprocally, the expression of a bovine *Oct4*-GFP transgene was not restricted to the ICM in mouse blastocysts suggesting that Tcfap2c binding to the *Oct4* promoter is required for repression of *Oct4* in the TE of blastocysts.

Additionally, *Oct4* promoter luciferase assays have yielded conflicting results on the role of Tcfap2c in *Oct4* transcription. Berg and co-workers showed that Tcfap2c, in addition to Cdx2, could downregulate *Oct4* transcriptional activity in ES cells [12]. In contrast, another study observed only mild repression of *Oct4* in a Tcfap2c-mediated *Oct4* promoter luciferase assay [14]. Particularly, these studies relied on reporter assays in ES cells that are not equivalent to cleavage stage embryos [12], [14]. To date no functional analyses have been performed to test the biological role of Tcfap2c in *Oct4* regulation during the window of preimplantation development.

Here we report that neither Tcfap2c nor its closely related family member Tcfap2a, are required for repression of *Oct4* in the TE of mouse blastocysts. Our analysis reveals that an Oct4-Tcfap2c interaction exists during mouse preimplantation development and that Tcfap2c may act as a co-activator of *Oct4* transcription by interacting with Oct4 protein at the CR4 region.

## Materials and Methods

### Embryo culture and microinjection of siRNAs

Mouse embryo collection, culture, and microinjection were performed as described previously [15]. We injected 5–10 pl of siRNAs (siGenome; Dharmacon, Lafayette, CO, USA) into the cytoplasm of one-cell zygotes using a PL100 picoinjector (Harvard Apparatus, Hollistan, MA, USA) in all of our experiments. Depending on the experiment either 10 or 100 µM target siRNAs and scrambled siRNAs were used. siRNA sequences of *Tcfap2c* and scrambled control can be found in a previous study [15] and target sequences of *Cdx2*, *Tcfap2a*, *Oct4* were as follows: *Cdx2*: GAGUUUCACUUUAGUCGAU, GACGUGAGCAUGUAUCCUA, CGGCGAAACCUGUGCGAGU and CCGAAUACCACGCGCACCA. *Tcfap2a*: GUAGAAGACCCGGGUAUUA, GGAGAGCGAAGUCUAAGAA, CCAGAUCAAACUGUAAUUA and GUAGCAUGUUAAACGAUUA. *Oct4*: GGUCAACGGUGUCCUCAAA, GAUAAUGGCCUACAAGAUG, GAGCGAAUGCGGAGGCUUA, and CAACGGGCCUUUCCUCAUC. All animal work conformed to the institutional guidelines and regulatory standards of Michigan State University (MSU). This study was approved by the Institutional Animal Care and Use Committee (IACUC) at MSU.

### Isolation of RNA, cDNA synthesis, and real-time reverse transcription PCR

Total RNA was isolated from a pool of embryos (at least 10/batch) at different stages of development using the PicoPure RNA isolation kit (Arcturus, Mountain View, CA, USA) and cDNA was synthesized by using SuperScript II reverse transcriptase (Invitrogen, Carlsbad, CA, USA). Real-time PCR was carried out on a StepOnePlus real-time PCR system (Applied Biosystems, Foster City, CA, USA) using TaqMan Probes (Applied Biosystems) for *Tcfap2c*, *Tcfap2a*, *Pou5f1*, *Nanog*, and *Cdx2*. *Ubtf* was used as an internal control as described previously [15].

### Immunocytochemistry (ICC) and *in situ* proximity ligation assay

ICC was conducted as previously described [15]. In brief, preimplantation embryos were fixed with 3.7% paraformaldehyde for 20 minutes, permeabilized with PBS containing 0.1% Tween 20 for 15 minutes, blocked with PBS containing 0.1% BSA for 1 hour at room temperature, and incubated with primary antibodies in blocking solution overnight at 4°C. Tcfap2c; rabbit polyclonal (Santa Cruz Biotechnology, Inc., Santa Cruz, CA, USA), Oct4; mouse monoclonal (Santa Cruz Biotechnology), Tcfap2a; rabbit polyclonal (Santa Cruz Biotechnology), Nanog; rabbit polyclonal (Cosmo Bio Co., Tokyo, Japan), and Cdx2; mouse monoclonal (San Ramon, CA, USA) were used as primary antibodies, and Alexa Fluor 488 and 594 (Molecular Probes, Eugene, OR, USA) were used as secondary antibodies. Embryos were then mounted in Vectashield containing DAPI (4, 6-diamidino-2-phenylindole; Vector Laboratories, Burlingame, CA, USA) and imaged using a spinning disc confocal module (CARV; Atto Bioscience, Rockville, MD, USA) with Metamorph software (Molecular Devices, Sunnyvale, CA, USA). Intensities of fluorescence detected and adjusted by Metamorph were quantified by using NIH Image J software (ImageJ 1.46r, http://imagej.nih.gov/ij/). For each sample, all the blastomeres of embryos were examined and the experiment was carried out at least five times. For Duolink proximity ligation assay (PLA), embryos were fixed, permeabilized and treated with primary antibodies as seen in ICC, followed by incubation with PLA probes, ligation, and amplification according to manufacturer’s protocol (Olink biosciences, Uppsala, Sweden).

### Chromatin immunoprecipitation (ChIP)-qPCR assay

To investigate enrichment of TFs on CR4 and CR1 regions of *Oct4*, we used a Cdx2 inducible ES cell line that differentiates into TS-like cells after Cdx2 induction by removal of doxycycline (Coriell Cell repositories, Camden, NJ, USA). ChIP was conducted as previously described [7]. In brief, ChIP was performed using two different types of cells, uninduced Cdx2 inducible ES cells (0 h) and induced TS-like cells (96 h after induction), and 5μg of antibodies including Tcfap2c (Santa Cruz Biotechnology), Tcfap2a (Santa Cruz Biotechnology), Oct4 (Santa Cruz Biotechnology), and a rabbit IgG (Millipore). ChIP samples were quantified by qPCR (SYBR green Master Mix; Applied Biosystems) using the following *Oct4* primers; 5′-TGAACTGTGGTGGAGAGTGC-3′(CR4 forward), 5′-AGGAAGGGCTAGGACGAGAG-3′(CR4 reverse), 5′-TAGGTGAGCCGTCTTTCCAC-3′(CR1/TSS forward), 5′-GCTTAGCCAGGTTCGAGGAT-3′(CR1/TSS reverse). ChIP-qPCR data were normalized using the percent input method.

### Statistical analysis

Data were analyzed by analysis of variance (ANOVA) using INSTAT3 (GraphPad Software, San Diego, CA, USA). The data are presented as mean ± s.e.m. (standard error mean). *P* values <0.05 were considered statistically significant differences unless otherwise stated.

## Results

### Depletion of Tcfap2c downregulates *Oct4* expression during the 8 cell-to-morula transition

**Figure 1:**
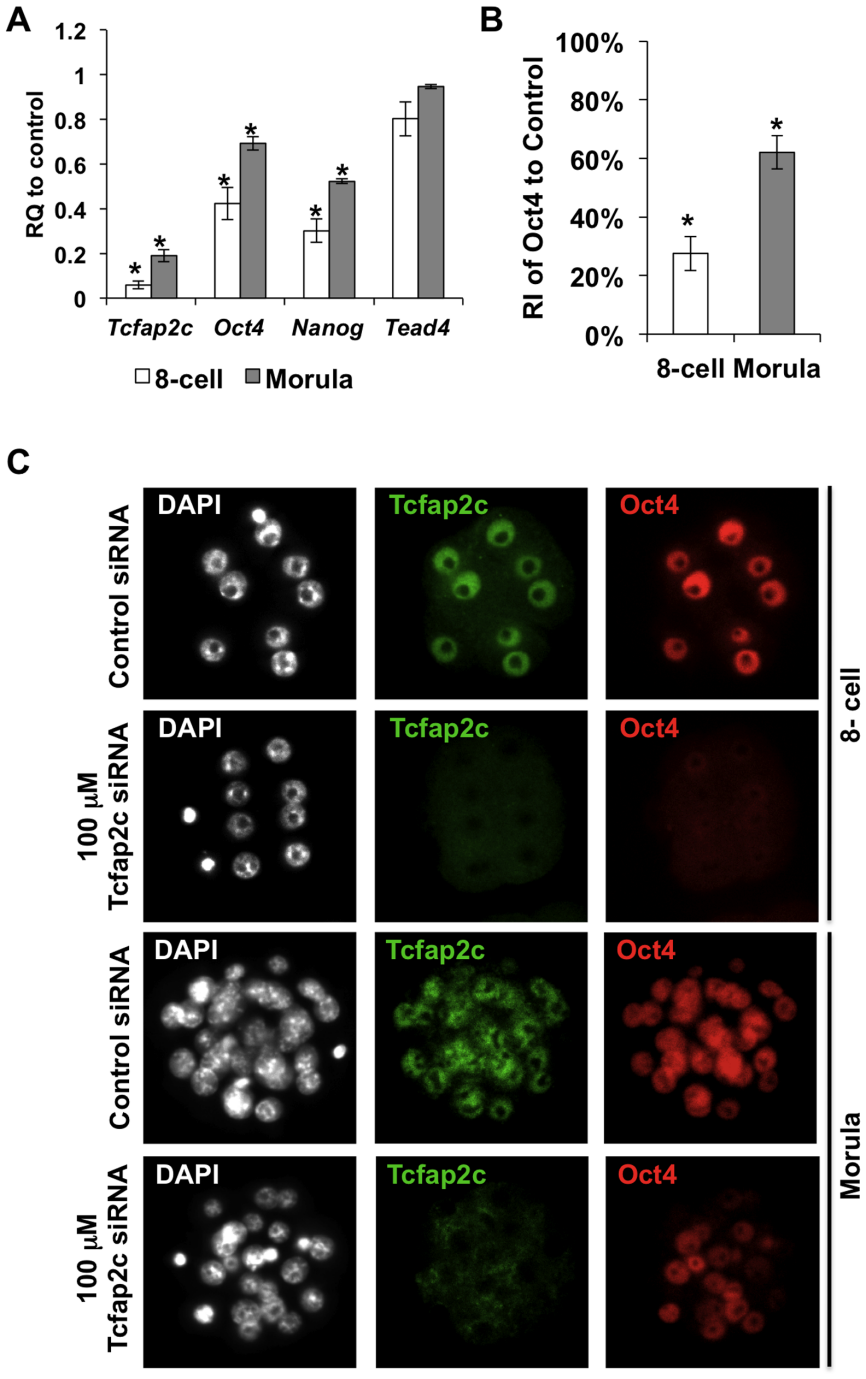
Depletion of Tcfap2c downregulates *Oct4* expression during preimplantation development. (A) Validation of siRNA-mediated knockdown (KD) of *Tcfap2c* transcripts and relative quantification of *Oct4*, *Nanog*, and *Tead4* transcripts in 8-cell embryos and morulae by qRT-PCR; RQ (Relative Quantification). Three to four biological replicates with 10–20 embryos per replicate were used. (B) Average levels of pixel intensities of Oct4-fluorescent signal in the nuclei of Tcfap2c KD 8-cell embryos and morulae were determined with the ImageJ software and compared to those of control (RI; relative intensities). Three biological replicates with five embryos per replicate were used to measure fluorescence intensities of nuclei in control and Tcfap2c KD embryos (8-cell stage = 120 nuclei/group; morula stage = 400 nuclei/group). (C) Protein expression and localization of Tcfap2c and Oct4 in Tcfap2c KD and control embryos. Error bars represent mean ± s.e.m. Asterisk symbol indicates P<0.05(*) compared with the control group.

During preimplantation development Oct4 and Tcfap2c are highly expressed and localized to the nuclei of 8-cell embryos and morulae [5], [8], [15]. To address the biological role of Tcfap2c in *Oct4* regulation during these stages of development we employed an RNA interference (RNAi) approach. The specificity of the *Tcfap2c* siRNA targeting sequences utilized in these experiments were previously documented [15]. Moreover, the effects of *Tcfap2c* siRNA on preimplantation development to the morula stage and cell numbers have been described previously [15]. We injected 100 µM *Tcfap2c* or control siRNA into fertilized one-cell embryos and cultured them *in vitro* for 2 to 3 days. At the 8-cell and morula stage embryos were evaluated by qRT-PCR and ICC, followed by relative quantification of the amount of Oct4 protein using Image-J software (Figure 1). Injection of 100 µM *Tcfap2c* siRNA triggered a 94 and 82% reduction in *Tcfap2c* transcripts at the 8-cell and morula stage, respectively (Figure 1A). Remarkably, *Oct4* transcript levels were significantly reduced in both 8-cell embryos and morulae (57.7% and 30.8%, respectively; *P*<0.05). Likewise, expression of *Nanog* was markedly reduced at these stages. The effects of *Tcfap2c* siRNA on downregulation of pluripotency gene expression did not appear to be related to a general shutdown in transcription as the expression of *Tead4* was not affected in these embryos (Figure 1A). Consistent with the decrease in transcripts Oct4 protein levels were reduced at both stages (Figure 1B, 1C; *P*<0.05). To test whether Tcfap2c regulates *Oct4* expression in a dose dependent manner, we microinjected a 10-fold lower (10 µM) concentration of *Tcfap2c* siRNA and evaluated *Oct4* transcripts and protein at the morula stage (Fig. S1). At this concentration a dose dependent decrease in *Oct4* transcripts and protein was observed. Collectively, these data suggest that Tcfap2c may positively regulate *Oct4* expression prior to blastocyst formation in mouse embryos.

### *Oct4* expression is properly downregulated in the TE of Tcfap2c KD blastocysts

**Figure 2:**
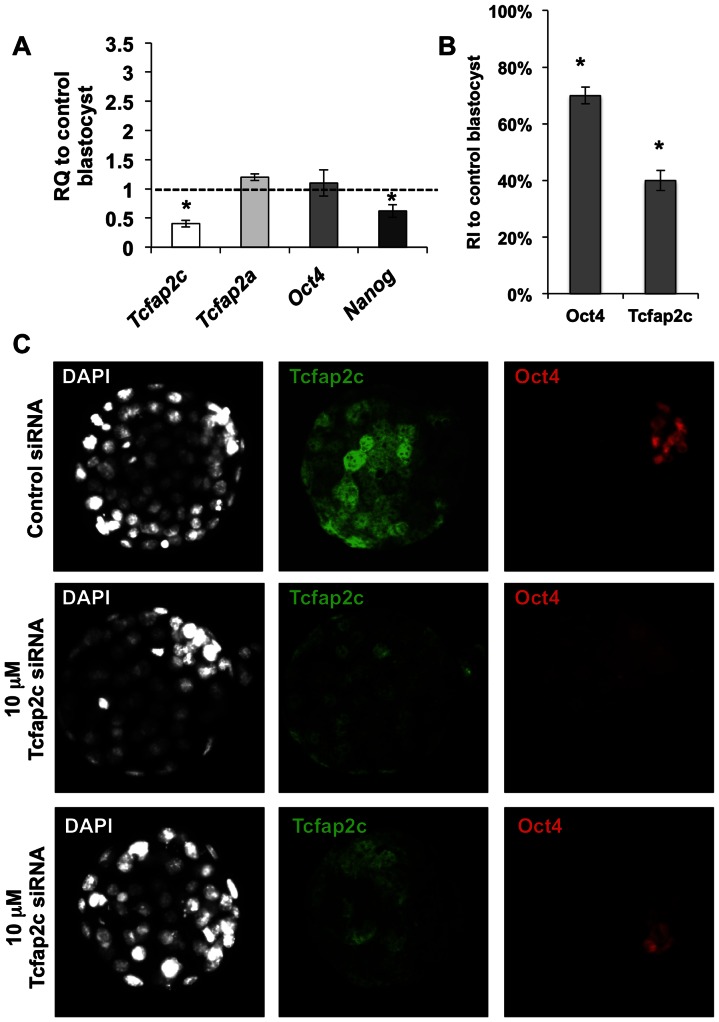
Tcfap2c is not required for restriction of *Oct4* expression in the blastocyst ICM. (A) Validation of siRNA-mediated KD of *Tcfap2c* transcripts and relative quantification of *Tcfap2a*, *Oct4*, and *Nanog* transcripts in blastocysts by qRT-PCR; RQ (Relative Quantification). Three to four biological replicates with at least 10 embryos per replicate were used. (B) Average levels of pixel intensities of Oct4 and Tcfap2c fluorescent signals in Tcfap2c KD blastocysts were determined with the ImageJ software and compared to those of control (RI; relative intensities). Three biological replicates with at least five embryos per replicate were used to measure the intensities of Oct4 and Tcfap2c in nuclei of control and Tcfap2c KD blastocysts. (C) Two representative images of *Oct4* expression in Tcfap2c KD blastocysts. Error bars represent mean ± s.e.m. Asterisk symbol indicates P<0.05(*) compared with the control group.

**Figure 3:**
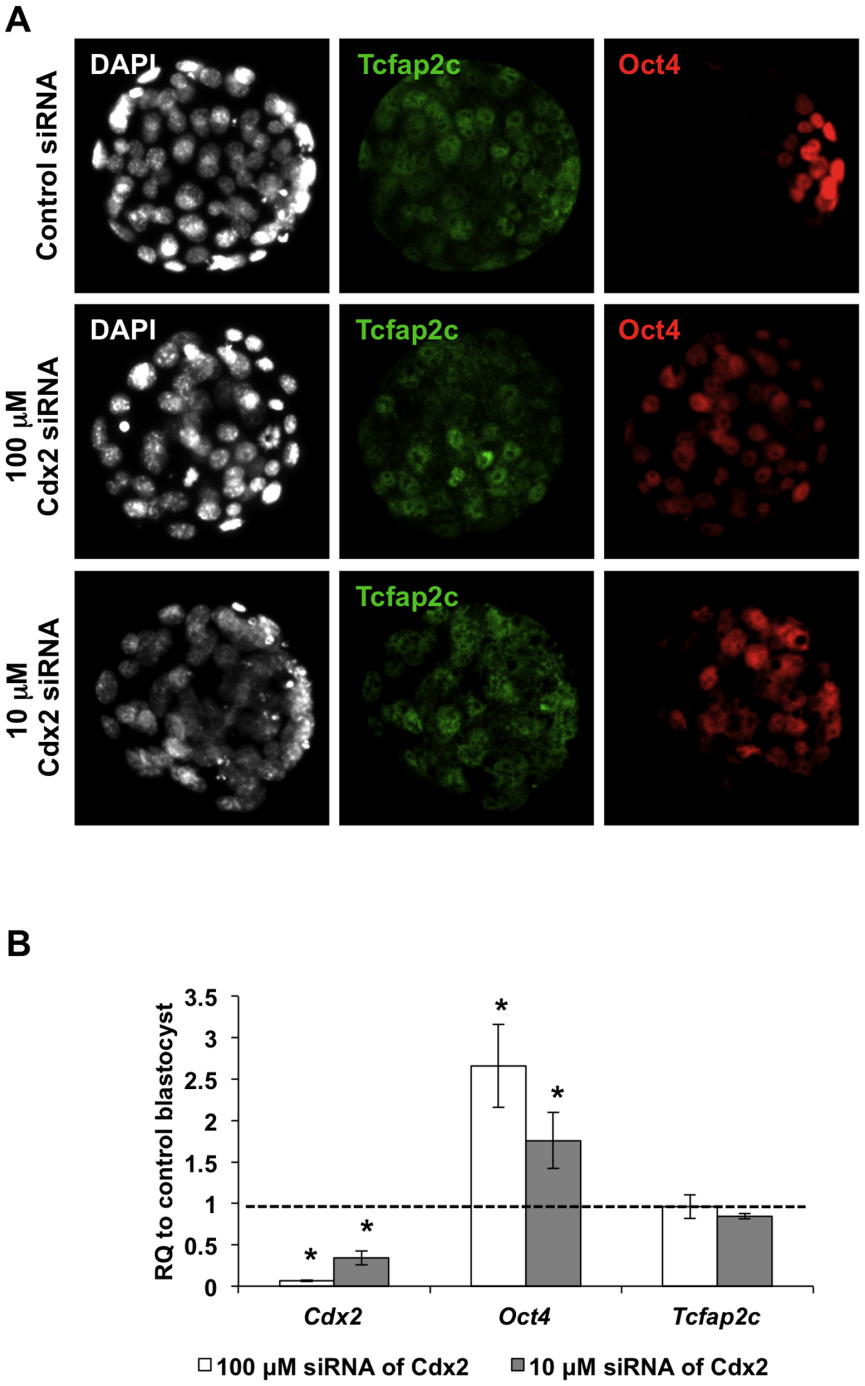
Depletion of Cdx2 inhibits *Oct4* repression and restriction to the ICM. (A) Expression and localization of Tcfap2c and Oct4 in Cdx2 KD (100 or 10 µM *Cdx2* siRNA) and control blastocysts. (B) Validation of siRNA-mediated KD of *Cdx2* transcripts (100 or 10 µM *Cdx2* siRNA), and expression of *Oct4* and *Tcfap2c* in blastocysts by qRT-PCR; RQ (Relative Quantification). Three biological replicates with at least 10 embryos per replicate were used. Error bars represent mean ± s.e.m. Asterisk symbol indicates P<0.05(*) compared with the control group.

Recently, we reported that Tcfap2c is critical for blastocyst formation in mice [15]. To elucidate the role of Tcfap2c in *Oct4* regulation at the blastocyst stage a 10-fold lower dose of *Tcfap2c* siRNA was utilized. Sixty-eight percent of zygotes injected with 10 µM *Tcfap2c* siRNA developed to the blastocyst stage (Fig. S2). Importantly, under this condition there was a 60% reduction in *Tcfap2c* transcripts and a corresponding decrease in protein (Figure 2; *P*<0.05). The total cell numbers in Tcfap2c KD and control blastocysts was 65±2.4 and 78.2±3.8, respectively (*P*<0.05). Next we compared the levels of *Oct4* transcripts and protein in Tcfap2c KD blastocysts with those in control blastocysts. To avoid any bias in potentially different levels of *Oct4* expression in different sized blastocysts we selected morphologically similar blastocysts at 120 hph (Fig. S2). As shown in Figure 2A, downregulation of Tcfap2c had no major impact on the levels of *Oct4* transcripts (*P*>0.05). Moreover, ICC analysis revealed that *Oct4* expression was properly localized to the ICM, but was slightly reduced (Figure 2B, 2C; *P*<0.05). In addition, the expression of *Nanog* and *Tcfap2a* were assessed. *Nanog* transcripts were reduced (*P*<0.05) while *Tcfap2a* transcripts were unaffected (Figure 2A; *P*>0.05). Consistent with Oct4 restriction, *Nanog* expression was confined to the ICM in Tcfap2c KD blastocysts (Fig. S3). In contrast, depletion of Cdx2 via injection of 10 or 100 µM *Cdx2* siRNA, resulted in widespread expression of *Oct4* in the blastocyst TE and a dose dependent increase in *Oct4* transcripts (Figure 3A, 3B; *P*<0.05). Interestingly, there was no difference in the levels of Tcfap2c between Cdx2 KD and control blastocysts (*P*>0.05), suggesting that Cdx2 does not regulate *Tcfap2c* expression in mouse blastocysts. Furthermore, such findings suggest that in the presence of decreased levels of Cdx2, other factors such as Tcfap2c do not compensate and repress *Oct4* expression in the TE lineage.

To further assess the biological function of Tcfap proteins in *Oct4* repression during blastocyst formation we focused on Tcfap2a. *Tcfap2a* is expressed at the late blastocyst stage and combined knockout of zygotic *Tcfap2c* and *Tcfap2a* results in developmental arrest shortly after implantation [16]. Recently, Berg and co-workers reported that Tcfap2a can act as a transcriptional repressor in *Oct4* promoter luciferase assays [12]. However, the biological function of Tcfap2a during preimplantation development was not evaluated. We first determined the developmental expression of *Tcfap2a* during blastocyst formation. qRT-PCR and ICC analysis revealed that expression of *Tcfap2a* was low in 8-cell embryos and morulae and became highly expressed in blastocysts when Oct4 was restricted to the ICM (Fig. S4). To test whether Tcfap2a was required for *Oct4* regulation and embryo development, one-cell embryos were injected with 100 µM *Tcfap2a* siRNA. Depletion of Tcfap2a had no effect on blastocyst formation or *Oct4* and *Nanog* expression (Figs. S2 and S5). Moreover, combined depletion of Tcfap2a and Tcfap2c (double KD) had no effect on the expression and restriction of Oct4 in blastocysts (Fig. S5). Altogether, these results suggest that *Oct4* silencing in mouse blastocysts is primarily mediated by Cdx2 and not by Tcfap2c and Tcfap2a.

### Tcfap2c interacts with Oct4 prior to blastocyst formation

**Figure 4:**
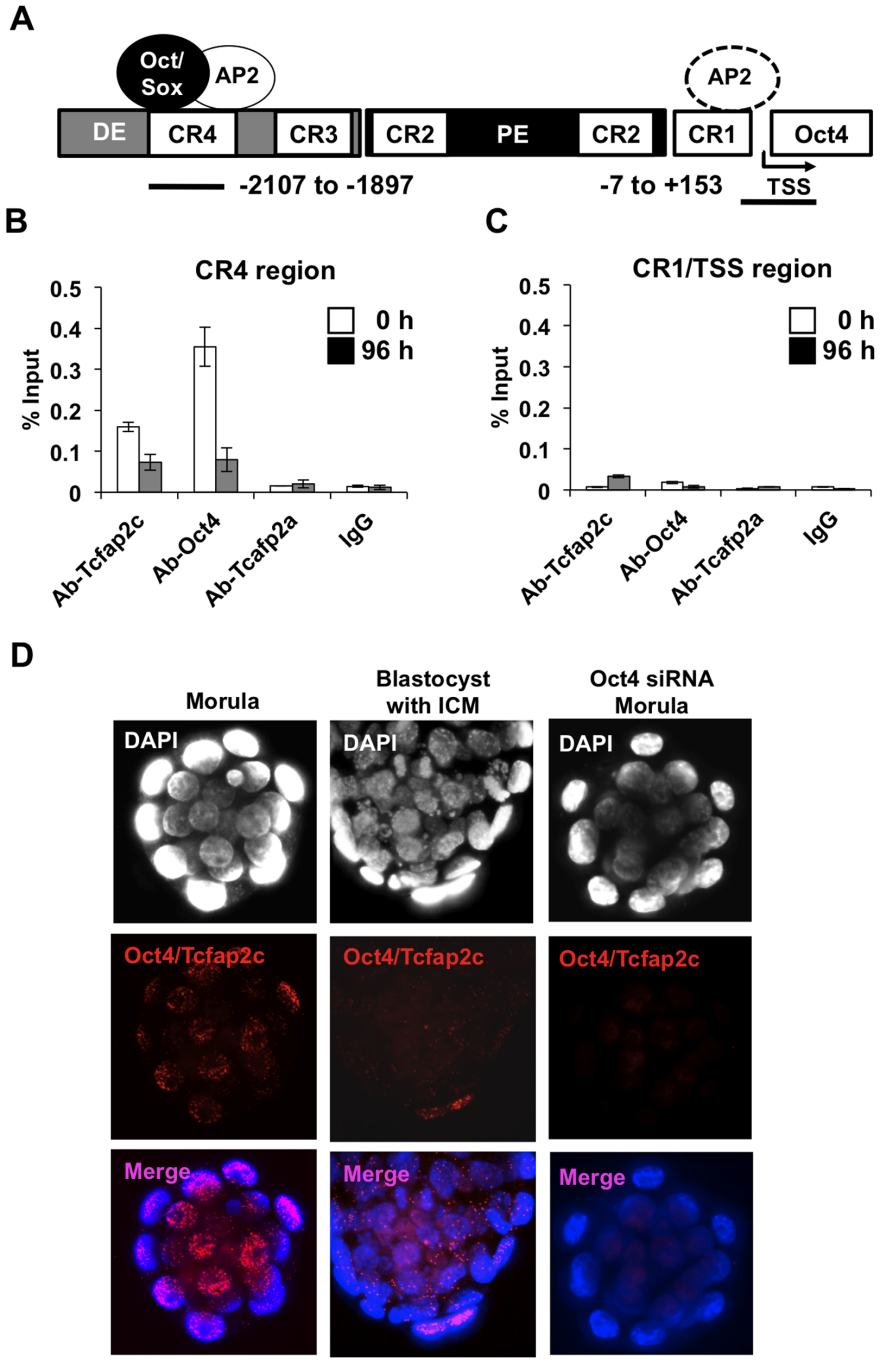
Tcfap2c and Oct4 interactions at the CR4 region of *Oct4* during early development. (A) Schematic diagram of the *Oct4* genomic region; DE (Distal Enhancer), PE (Proximal Enhancer), CR (Conserved Region), TSS (Transcription start site), Oct/Sox(Oct4/Sox2 binding site), AP2(Tcfap2 binding site); black line and numbers (amplified regions for ChIP-qPCR and sequences from TSS). (B) ChIP analysis of Tcfap2c, Oct4, Tcfap2a binding to CR4 in uninduced ES cells (0 h) and Cdx2 induced TS-like cells (96 h). (C) ChIP analysis of Tcfap2c, Oct4, Tcfap2a binding to CR1/TSS region. (D) A proximity ligation assay (Duolink) in morulae and blastocysts. A subset of embryos were injected with *Oct4* siRNA to deplete Oct4. Red dotted fluorescence indicates sites of interaction of Oct4 and Tcfap2c. Two to three biological replicates were used. Error bars represent mean ± s.e.m.

Considering the aforementioned results in Tcfap2c KD embryos, we speculated that Tcfap2c may interact with Oct4 protein at the enhancer in the CR4 region to positively regulate its expression. To determine whether Tcfap2c can directly bind to the AP2 motif within the *Oct4* CR4 region we assessed recruitment of Tcfap2c, Oct4 or Tcfap2a to –2107 to –1897 upstream of the transcription start site (TSS) and –7 upstream to +153 downstream (CR1/TSS) from TSS of *Oct4* via ChIP analyses using a doxycycline controllable Cdx2-inducible ES cell line as a model system for the developing blastocyst trophectoderm [7], [15], [17]. Oct4 and Tcfap2c were strongly bound to the mouse CR4 region in uninduced ES cells (0 h). However, during differentiation into TS-like cells (96 h), when *Oct4* transcription was downregulated, the occupancy of Oct4 and Tcfap2c was lost (Figure 4A, 4B). We also examined CR1/TSS region containing a putative AP2 binding site analyzed by TRANSFAC and Explain 3.0, and found that reciprocal binding of Tcfap2c and Oct4 occurs in this region, although the enrichments were not significant compared to those in ES cells (Figure 4C). Tcfap2a which was previously proposed as a repressor of *Oct4* [12] was not recruited to the CR4 or CR1/TSS region in ES cells differentiating into TS-like cells (Figure 4B, 4C), suggesting Tcfap2a is dispensable for regulation of *Oct4* expression.

To test whether Oct4 and Tcfap2c interact during preimplantation development, we carried out a Duolink proximity ligation assay (PLA), a fluorescence-based method to assess protein-protein interactions. In agreement with the ChIP results above, red-fluorescent labeling was strongly detected in the nuclei of morulae when Tcfap2c and Oct4 are co-expressed, but was lost during blastocyst formation when the expression of *Oct4* and *Tcfap2c* become restricted to the ICM and TE, respectively (Figure 4D). To confirm that the observed signal was due to Oct4 and Tcfap2c interactions, a second group of embryos were injected with *Oct4* siRNA. Depletion of Oct4 resulted in a loss of red fluorescence confirming that Oct4 interacts with Tcfap2c prior to blastocyst formation (Figure 4D). These results demonstrate that Tcfap2c and Oct4 bind and may functionally interact at the CR4 region to positively regulate *Oct4* expression prior to blastocyst formation. Further investigation is necessary to confirm whether Tcfap2c is indeed a co-activator of *Oct4* expression during preimplantation development. Nevertheless, these data support the hypothesis that Tcfap2c is not a major repressor of *Oct4* expression in the TE lineage during blastocyst formation.

## Discussion

Recent work in a bovine model demonstrated that Cdx2 was not sufficient to downregulate *Oct4* expression in the TE lineage of blastocysts [12]. Comparative analysis of the bovine, human, and mouse *Oct4* promoters revealed that the mouse *Oct4* promoter contains a Tcfap2c binding motif in the CR4 region that is absent in both humans and cattle. However, functional studies were not conducted to test the proposed role for Tcfap2c in *Oct4* repression in mouse blastocysts [12]. To date several functional studies have demonstrated that Cdx2 is the predominant negative regulator of *Oct4* expression in mouse blastocysts. First, several labs have shown that depletion of Cdx2 by utilizing a knockout or RNAi approach resulted in the failure to repress *Oct4* expression in the TE lineage of blastocysts [6], [7], [8]. Second, forced expression of *Cdx2* in mouse ES cells downregulates *Oct4* expression via promoter and protein interactions [4]. Third, Cdx2 cooperates with the chromatin remodeling protein Brg1 at the *Oct4* promoter enhancer region to downregulate *Oct4* transcription in the TE during mouse early embryogenesis [7]. Altogether, these studies demonstrate that Cdx2 is a key regulator of *Oct4* expression and/or function during mouse TE development.

To address the biological role of Tcfap2c in *Oct4* regulation we adopted an RNAi approach instead of a more traditional gene knockout strategy because of its ability to reduce Tcfap2c in a dose-dependent manner and to circumvent embryonic lethality. Recently, we demonstrated that Tcfap2c is important for blastocyst formation in mice [15]. Thus the combined knockout of maternal and zygotic *Tcfap2c* would likely result in embryonic lethality prior to the blastocyst stage preventing analysis at this stage. Accordingly we determined that RNAi-mediated downregulation of Tcfap2c and/or Tcfap2a had no effect on *Oct4* repression at the blastocyst stage, whereas, injection of similar concentrations of *Cdx2* siRNA that caused a comparable level of knockdown, resulted in a significant increase in *Oct4* transcripts and widespread expression of Oct4 protein in mouse blastocysts. It is possible that the level of Tcfap2c and Tcfap2a knockdown that was achieved via RNAi was not sufficient to elicit an effect on *Oct4* expression, however, we feel this is not the case since a similar reduction in Cdx2 had a dramatic effect on *Oct4* transcription. Alternatively, it is possible that Tcfap2c cooperates with Cdx2 to mediate *Oct4* repression at the mRNA and protein level. To address this possibility we performed double knockdowns of Tcfap2c and Cdx2 in preimplantation embryos (data not shown). Unfortunately, combined depletion of both factors resulted in embryonic lethality at the morula stage. In future experiments it will be important to deplete Tcfap2c in Cdx2-inducible ES cells to test whether Tcfap2c is required for Cdx2-mediated silencing of *Oct4* expression. Altogether, our results suggest that Tcfap2c and/or Tcfap2a are likely not involved in Cdx2-mediated repression of *Oct4* expression in mouse embryos.

Our most interesting finding was the effect of *Tcfap2c* siRNA on *Oct4* expression at the 8-cell and morula stage. Prior to blastocyst formation *Oct4* is uniformly expressed in all blastomeres and during blastocyst formation *Oct4* expression becomes restricted to the ICM [5]. Depletion of Tcfap2c caused a significant decrease in *Oct4* transcripts and protein levels at the 8-cell and morula stage. Likewise reduced levels of Oct4 protein were observed in the ICM of Tcfap2c KD blastocysts demonstrating that the effects of Tcfap2c on *Oct4* expression at the 8-cell and morula stage are carried over to the blastocyst stage. To test whether Tcfap2c could interact with Oct4 protein prior to blastocyst formation two experiments were conducted. In uninduced Cdx2-inducible ES cells we found that both Oct4 and Tcfap2c bind to the *Oct4* CR4 region. In contrast, following Cdx2 induction when *Oct4* expression was silenced, Tcfap2c binding was lost at the CR4 region. Furthermore, in morulae Tcfap2c and Oct4 protein interactions were detected, however, during blastocyst formation those interactions were lost. Combined these observations suggest that Tcfap2c may functionally interact with the enhancer in the *Oct4* CR4 region to positively regulate *Oct4* expression during early embryogenesis. Additional experiments are necessary to confirm whether Tcfap2c is a bona fide co-activator of *Oct4* expression during preimplantation development. It will be important to ablate Tcfap2c in a stage-specific manner to elucidate the precise role of Tcfap2c in *Oct4* regulation at the blastocyst stage; the reduced levels of Oct4 at the 8-cell and morula stage in Tcfap2c KD embryos may have shifted the equilibrium between Oct4 and Cdx2 levels causing downregulation of *Oct4* expression in the TE lineage even in the presence of decreased levels of Tcfap2c.

In conclusion, functional studies in a mouse model provide strong evidence that Tcfap2c and Tcfap2a are not required for *Oct4* repression in the TE of mouse blastocysts. These findings support the notion that Cdx2 is the primary repressor of *Oct4* expression in the blastocyst TE. Furthermore, these data demonstrate that differences in the developmental timing of *Oct4* repression in mouse and cattle blastocysts is most likely not related to Tcfap2c, but is likely due to other intrinsic factors.

## Supporting Information

Figure S1 **Effects of 10** µ**M** ***Tcfap2c*** **siRNA on expression and subcellular localization of Oct4 in Tcfap2c KD morulae. (A)** Protein expression and localization of Oct4 in Tcfap2c KD (10 µM siRNA) and control embryos. (B) Validation of 10 µM siRNA-mediated KD of *Tcfap2c* transcripts and RQ of *Oct4*, *Nanog* and *Tead4* transcripts in morula by qRT-PCR. Asterisk symbol indicates P<0.05(*) compared with the control group.
(TIF)

Figure S2 **siRNA-mediated knockdown of Tcfap2 family genes in mouse preimplantation embryos.** (A) Effect of 10 µM *Tcfap2c* siRNA (n = 85), 100 µM *Tcfap2a* siRNA (n = 51), or a combination of 10 µM *Tcfap2c* and 100 µM *Tcfap2a* siRNA (n = 49) on mouse preimplantation development. Blastocyst rates of each siRNA injected group were compared to those of control group (n = 55). At least three biological replicates were used. (B) Representative images of Tcfap2c KD (10 µM siRNA) and control blastocysts at 120 hph (upper) and blastocysts selected for qRT-PCR (bottom). Error bars represent mean ± s.e.m. Asterisk symbol indicates P<0.05(*) compared to the control group.
(TIF)

Figure S3 **Expression and subcellular localization of Oct4 and Nanog in Tcfap2c KD blastocysts.** Protein expression and localization of Oct4 and Nanog in Tcfap2c KD and control blastocysts. A total of three biological replicates were utilized.
(TIF)

Figure S4 **Developmental expression of Tcfap2a during mouse preimplantation development.** (A) qRT-PCR analysis of Tcfap2a transcripts in MII oocytes, 2-cells, 8-cells, morulae, and blastocysts; expression levels from each stage were normalized to exogenous GFP and are relative to MII oocytes. RQ (Relative Quantification). 10 embryos per stage were collected and two technical and three biological replications were performed. (B) ICC analysis revealed that expression of Tcfap2a was barely detectable until the morula stage and was enriched in the TE of blastocysts. Oct4 staining denotes the ICM. Error bars represent mean ± s.e.m.
(TIF)

Figure S5 **Depletion of Tcfap2a or combined depletion of Tcfap2a and Tcfap2c does not affect Oct4 restriction in blastocysts.** (A) qRT-PCR analysis of Oct4, Nanog, Tcfap2a, and Tcfap2c in Tcfap2a KD blastocysts (100 µM *Tcfap2a* siRNA). (B) Expression and subcellular localization of Oct4 and Tcfap2a in control blastocysts (top), Tcfap2a KD blastocysts (middle), and Tcfap2a and Tcfap2c double KD blastocysts (bottom; Tcfap2a/c KD). A total of three biological replications were performed. Error bars represent mean ± s.e.m. Asterisk symbol indicates P<0.05(*) compared with the control group.
(TIF)
