# Supplementary figures and images for: Evidence that Transcription Factor AP-2γ Is Not Required for Oct4 Repression in Mouse Blastocysts
Source: PLoS One. 2013 May 31;8(5):e65771. doi: 10.1371/journal.pone.0065771 (PMC3669238; doi:10.1371/journal.pone.0065771)

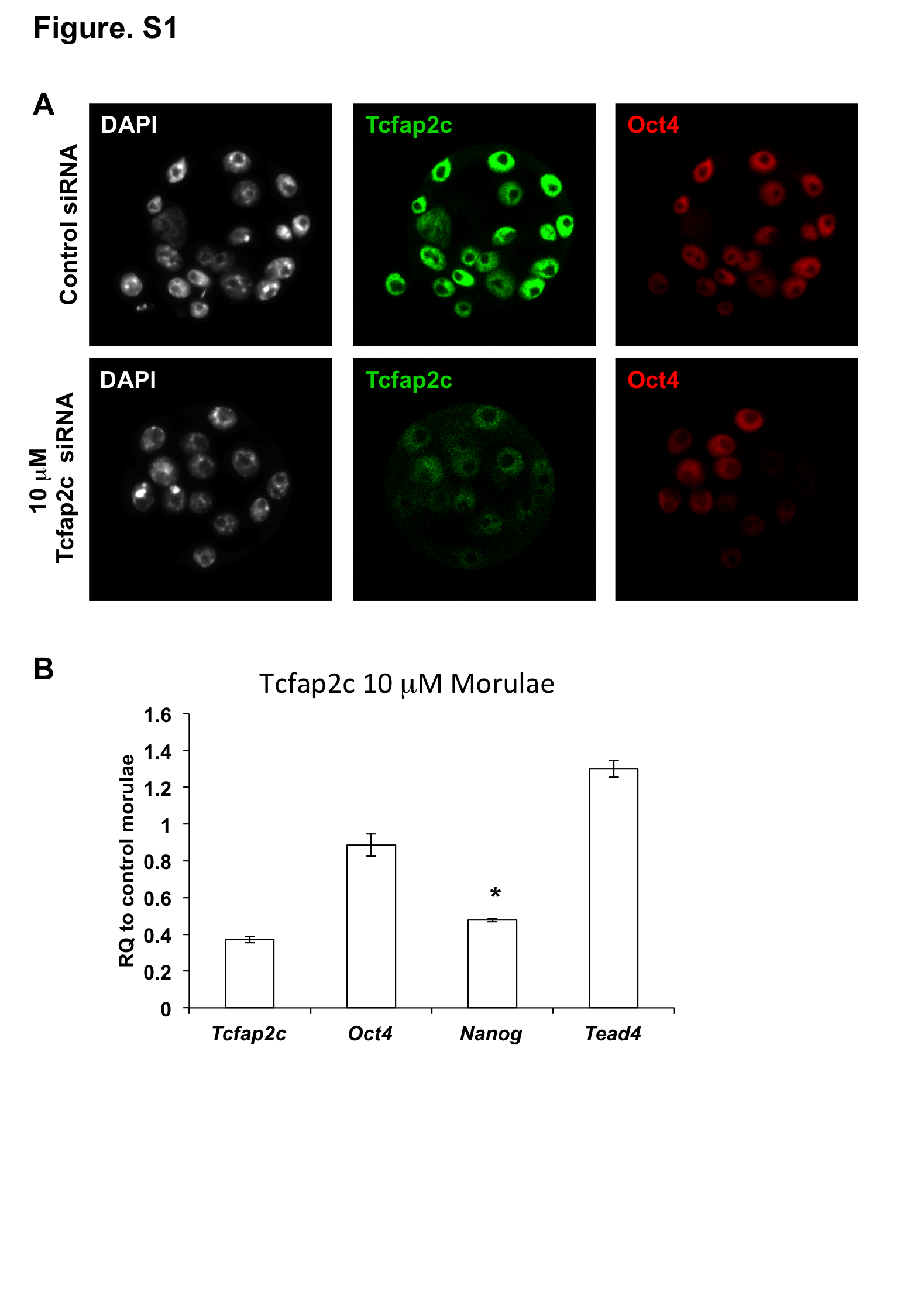

Supplement: Figure S1 — Effects of 10 µM Tcfap2c siRNA on expression and subcellular localization of Oct4 in Tcfap2c KD morulae. (A) Protein expression and localization of Oct4 in Tcfap2c KD (10 µM siRNA) and control embryos. (B) Validation of 10 µM siRNA-mediated KD of Tcfap2c transcripts and RQ of Oct4, Nanog and Tead4 transcripts in morula by qRT-PCR. Asterisk symbol indicates P<0.05(*) compared with the control group. (TIF) [file pone.0065771.s001.tif]

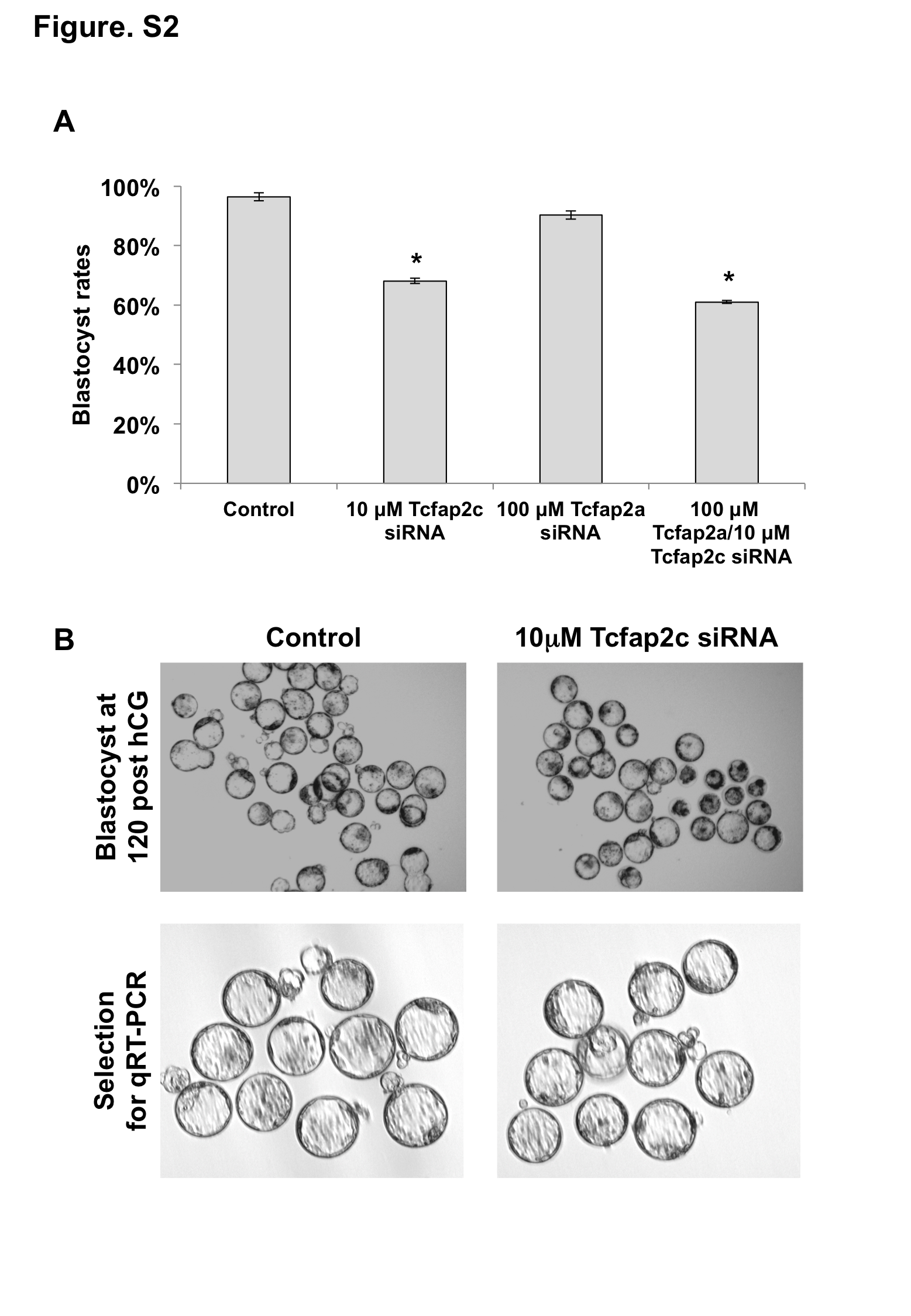

Supplement: Figure S2 — siRNA-mediated knockdown of Tcfap2 family genes in mouse preimplantation embryos. (A) Effect of 10 µM Tcfap2c siRNA (n = 85), 100 µM Tcfap2a siRNA (n = 51), or a combination of 10 µM Tcfap2c and 100 µM Tcfap2a siRNA (n = 49) on mouse preimplantation development. Blastocyst rates of each siRNA injected group were compared to those of control group (n = 55). At least three biological replicates were used. (B) Representative images of Tcfap2c KD (10 µM siRNA) and control blastocysts at 120 hph (upper) and blastocysts selected for qRT-PCR (bottom). Error bars represent mean ± s.e.m. Asterisk symbol indicates P<0.05(*) compared to the control group. (TIF) [file pone.0065771.s002.tif]

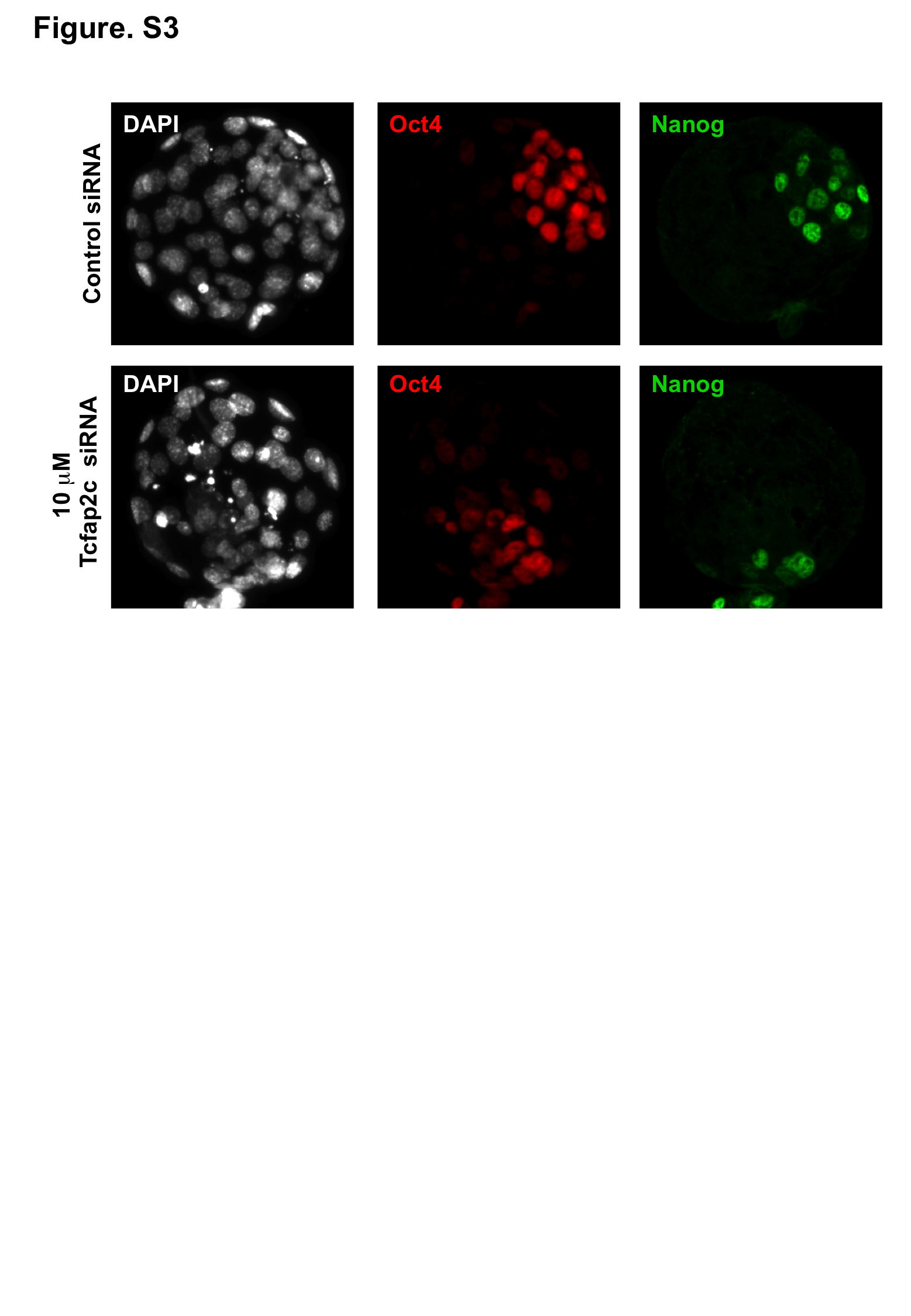

Supplement: Figure S3 — Expression and subcellular localization of Oct4 and Nanog in Tcfap2c KD blastocysts. Protein expression and localization of Oct4 and Nanog in Tcfap2c KD and control blastocysts. A total of three biological replicates were utilized. (TIF) [file pone.0065771.s003.tif]

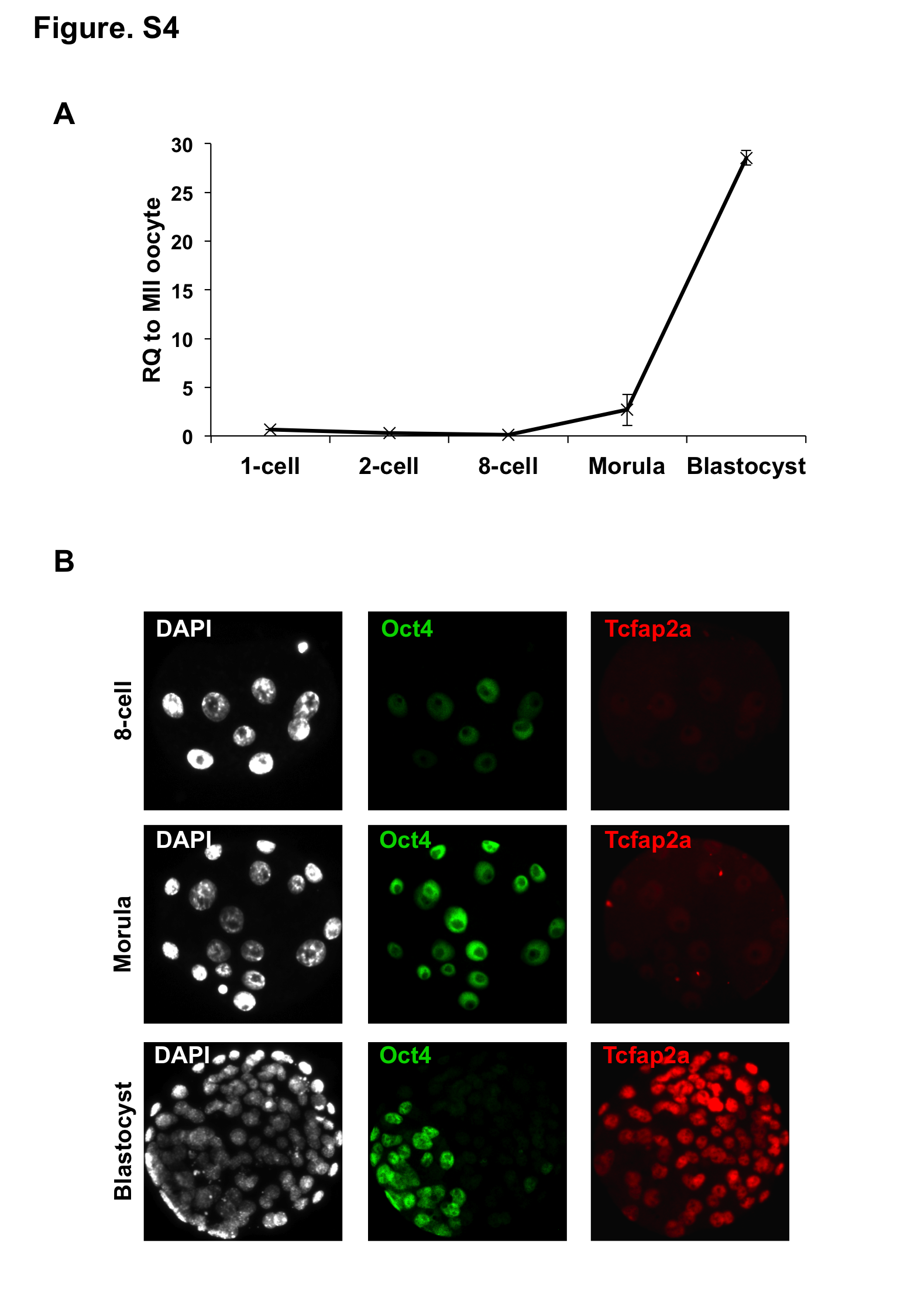

Supplement: Figure S4 — Developmental expression of Tcfap2a during mouse preimplantation development. (A) qRT-PCR analysis of Tcfap2a transcripts in MII oocytes, 2-cells, 8-cells, morulae, and blastocysts; expression levels from each stage were normalized to exogenous GFP and are relative to MII oocytes. RQ (Relative Quantification). 10 embryos per stage were collected and two technical and three biological replications were performed. (B) ICC analysis revealed that expression of Tcfap2a was barely detectable until the morula stage and was enriched in the TE of blastocysts. Oct4 staining denotes the ICM. Error bars represent mean ± s.e.m. (TIF) [file pone.0065771.s004.tif]

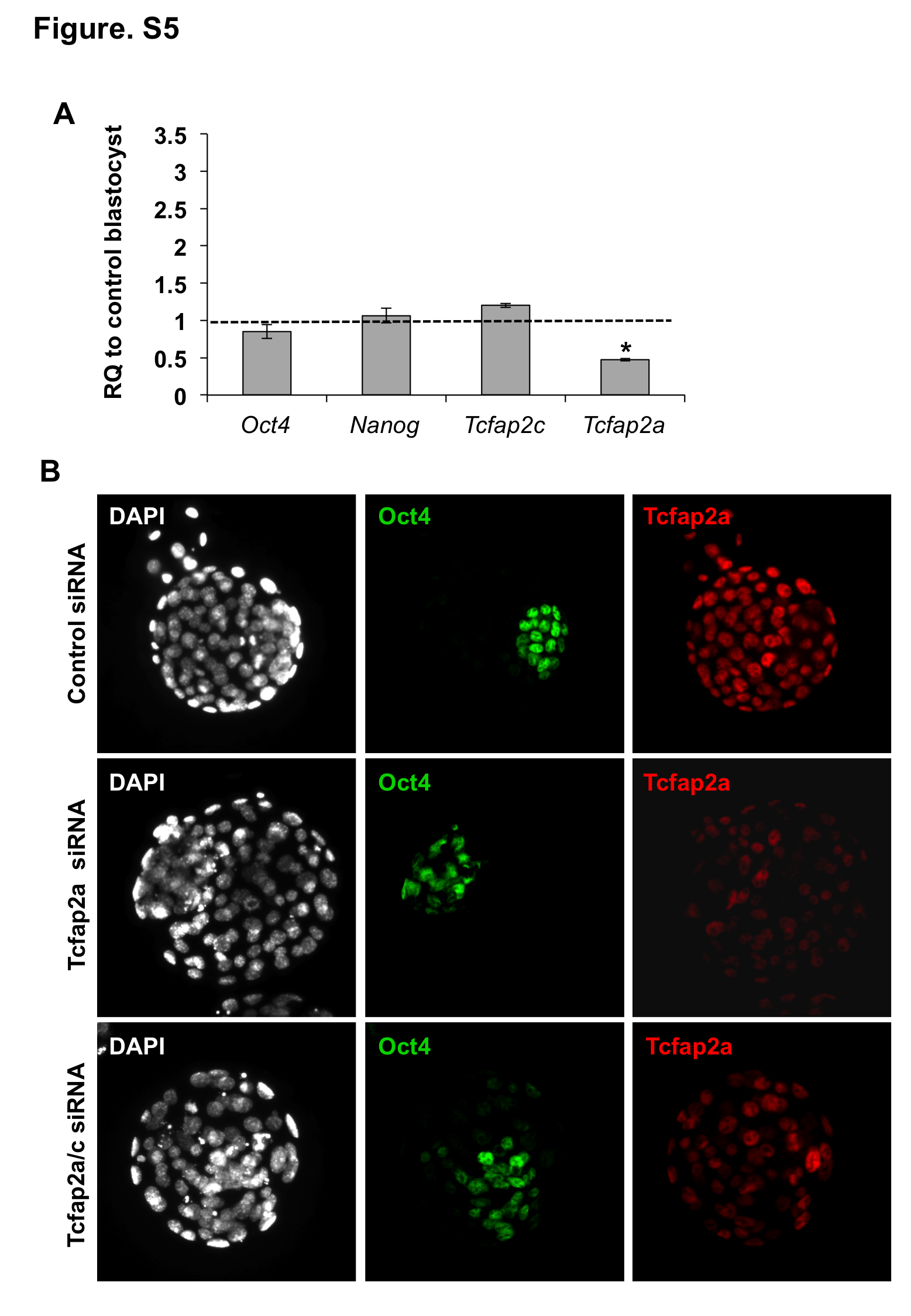

Supplement: Figure S5 — Depletion of Tcfap2a or combined depletion of Tcfap2a and Tcfap2c does not affect Oct4 restriction in blastocysts. (A) qRT-PCR analysis of Oct4, Nanog, Tcfap2a, and Tcfap2c in Tcfap2a KD blastocysts (100 µM Tcfap2a siRNA). (B) Expression and subcellular localization of Oct4 and Tcfap2a in control blastocysts (top), Tcfap2a KD blastocysts (middle), and Tcfap2a and Tcfap2c double KD blastocysts (bottom; Tcfap2a/c KD). A total of three biological replications were performed. Error bars represent mean ± s.e.m. Asterisk symbol indicates P<0.05(*) compared with the control group. (TIF) [file pone.0065771.s005.tif]
